# Multispecies genome-wide analysis defines the MAP3K gene family in *Gossypium hirsutum* and reveals conserved family expansions

**DOI:** 10.1186/s12859-019-2624-9

**Published:** 2019-03-14

**Authors:** Norbert Bokros, Sorina C. Popescu, George V. Popescu

**Affiliations:** 1 Department of Biochemistry, Molecular Biology, Plant Pathology and Entomology, Mississippi State University, Mississippi State, MS 39762 USA; 2 Institute for Genomics, Biocomputing and Bioengineering, Mississippi State University, Mississippi State, MS 39762 USA; 3 The National Institute for Laser, Plasma & Radiation Physics, Bucharest, Romania

**Keywords:** MAP3K gene family, HMM-profile gene search, Orthologous gene search, Sequence motif conservation, Phylogenetic analysis, Gene duplication, Gene collinearity, *Gossypium hirsutum*

## Abstract

**Background:**

Gene families are sets of structurally and evolutionarily related genes – in one or multiple species – that typically share a conserved biological function. As such, the identification and subsequent analyses of entire gene families are widely employed in the fields of evolutionary and functional genomics of both well established and newly sequenced plant genomes. Currently, plant gene families are typically identified using one of two major ways: 1) HMM-profile based searches using models built on *Arabidopsis thaliana* genes or 2) coding sequence homology searches using curated databases. Integrated databases containing functionally annotated genes and gene families have been developed for model organisms and several important crops; however, a comprehensive methodology for gene family annotation is currently lacking, preventing automated annotation of newly sequenced genomes.

**Results:**

This paper proposes a combined measure of homology identification, motif conservation, phylogenomic and integrated gene expression analyses to define gene family structures in multiple plant species. The MAP3K gene families in seven plant species, including two currently unexamined species *Gossypium hirsutum,* and *Zostera marina*, were characterized to reveal new insights into their collective function and evolution and demonstrate the effectiveness of our novel methodology.

**Conclusion:**

Compared with recent reports, this methodology performs significantly better for the identification and analysis of gene family members in several monocots/dicots, diploid as well as polyploid plant species.

**Electronic supplementary material:**

The online version of this article (10.1186/s12859-019-2624-9) contains supplementary material, which is available to authorized users.

## Background

Mitogen-activated protein kinase (MAPK) cascades are conserved signal transduction pathways with important functions in plant growth, development, and response to environmental stresses in all eukaryotic organisms. Consequently, the identification of their members – MAP3Ks, MAP2Ks, and MAPKs - is critical to a complete understanding of how plants respond to their increasingly challenging environments. Presently, the MAP3K, MAP2K, and MAPK gene families have already been characterized in numerous plant species including Arabidopsis, strawberry, maize, canola, diploid cotton, rice, barrelclover, tomato, soybean, and grape [1–10]. While MAP3Ks represent the largest, most divergent, and a poorly characterized component of the signaling cascade, continued research into how MAP3Ks function has yielded a wealth of data that has yet to be integrated into a much-needed refinement of MAP3K genomic architectures established over a decade ago [11].

As sequence data continues to accumulate for an ever increasing number of species, BLAST-based, HMM-based, and comparative homology-based searches have regularly been employed to identify entire gene families in a wide range of species. BLAST-based approaches have generally enjoyed the most popularity and involve using members of a known gene family in well-studied species to identify appropriate gene family members in a new organism of interest based on local sequence homology. Recently, HMM-based searches have been gaining popularity and display higher accuracy in gene family identifications compared to traditional BLAST-based approaches [12]. Instead of relying on individual sequences to query a database, HMM-based searches build a single probabilistic model of an entire gene family using a collection of previously validated sequences. Although both methods work well at identifying complete gene families, they also require extensive manual curation steps where hits are filtered to remove sequences that lack conserved sequence motifs or functional domains. While online databases such as Phytozome, PLAZA, and GreenPhylDB have been described as the highest performing gene family identification tool currently available [13], they often either include erroneously identified sequence hits, lack appropriate annotations necessary for accurate gene family identification, or exclude from analyses many newly sequenced species.

One of the central aims of this work is to refine the underlying architecture of the MAP3K gene family following the evolution of flowering plants. To this end, seven plant proteomes representative of the two major descendants of angiosperms, monocots, and dicots - were assembled and critically (re-) examined to identify and validate their collective MAP3K gene families. Of the five previously examined species, tomato, maize, and *Gossypium raimondii* relied on local BLAST searches using previously identified Arabidopsis, rice, and maize MAP3K sequences whereas for soybean HMM models built from a comprehensive collection of known MAP3Ks were utilized to identify new MAP3K genes. While these two methods work well at defining gene families, this study argues that a more integrative method utilizing orthogroup identification – resulting from the OrthoMCL workflow – combined with HMMsearches can be a reliable methodology for gene family classifications. Conserved motif analysis, phylogenetic analysis, and gene duplication/collinearity analysis can further be supplemented to improve the accuracy of identifying gene family structures and provide additional functional and evolutionary insights.

**Fig. 1:**
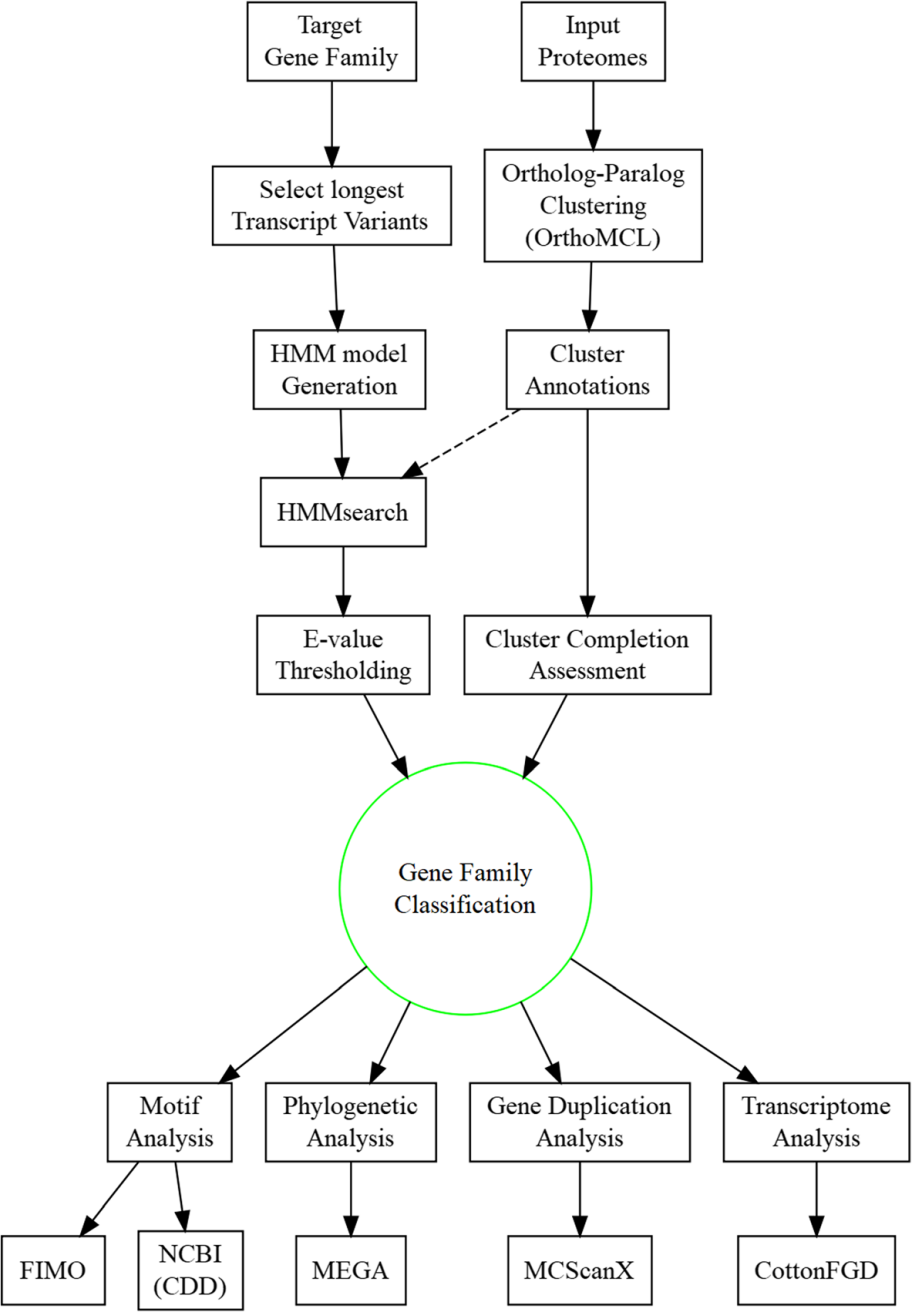
Flowchart illustrating steps for gene family identification and subsequent analysis

We describe here a sequential workflow where homology search is used first to decide on gene classification, followed by conserved motif analysis, phylogenetic and gene duplication analysis to define gene family evolution; a final step of integrating gene expression patterns with phylogeny offer additional functional insights and validation of gene family analysis. Figure 1 illustrates the workflow conducted in this study. Our focus here is to investigate the integration of homology search and phylogenetic analyses methods to define the first step towards a novel computational method for automated classification and analysis of gene families. We apply our methodology to identify the structure of the MAP3K gene family, a kinase family that includes conserved as well as expanded gene clades that are currently inadequately characterized in plants. The proposed methodology allowed for the identification of gene family clusters, filtered to include distinct, yet correctly homologous members (uniquely evolved singletons) while excluding hits which share ambiguous or distantly related homology as defined by similarity to newly built HMM models. In addition, our methodology allows for robust identification of gene family members in genomes that lack chromosome-scale genome assemblies or which are absent from online comparative homology databases.

## Results and discussion

### Cluster database construction

The proteomes of *Arabidopsis thaliana*, *Glycine max*, *Gossypium raimondii*, *G. hirsutum*, *Solanum lycopersicum*, *Zea mays* and *Zostera marina* were gathered from Phytozome and clustered into orthogroups of orthologs and recent paralogs by OrthoMCL as described below in *Methods*. 382,192 proteins from the seven proteomes were clustered into 40,524 orthogroups, excluding singletons; 63,913 unclustered proteins (singletons) were appended to this dataset to generate a final set of 104,437 orthogroups. Table 1 below shows the representation of each species in the 40,524 major orthogroups.

**Table 1:** OrthoMCL output clusters

Species	Input Protein Sequences	Clusters Containing Species	Unique Clusters	Absent Clusters
*A. thaliana*	48,456	14,687 (36.24%)	1793	220
*G. max*	88,647	16,671 (41.14%)	2603	75
*G. hirsutum*	87,800	23,315 (57.53%)	1686	11
*G. raimondii*	77,267	23,016 (56.79%)	1274	18
*Z. mays*	88,760	19,891 (49.08%)	8115	520
*S. lycopersicum*	34,725	14,318 (35.33%)	1114	244
*Z. marina*	20,450	10,914 (26.93%)	507	1001

All transcript variants were retained during clustering. “Unique Clusters” depict clusters containing only sequences from a particular species, and “Absent Clusters” represent clusters missing sequences from a particular species. They are associated with orthogroups uniquely present or uniquely missing in a particular species of those examined. Singleton clusters are not included in these counts.

### MAP3K identification

MAP3Ks in *Arabidopsis thaliana* [1, 14], *Glycine max* [9], *Gossypium raimondii* [5], *Solanum lycopersicum* [8], and *Zea mays* [3] had previously been critically examined, while identification of MAP3Ks in *Gossypium hirsutum* and *Zostera marina* is still lacking. As described in Fig. 1, our pipeline uses two homology search methods to classify MAP3K genes from the seven target species. First, we used OrthoMCL to identify orthogroups as described in the previous section. By utilizing previously identified MAP3Ks, we uncovered MAP3K orthoclusters containing sequence hits for all seven examined species. Second, we used profile-HMM homology search to identify candidate MAP3Ks from all seven target species. HMM models were built using previously identified Arabidopsis MAP3K protein coding sequences (available in Additional file 1, HMMs) for each gene subfamily (ZIK, MEKK, RAF). The HMMsearch output (available in Additional file 1, HMM_results) and the cluster completion ratio (percentage of genes in an orthocluster above the HMMsearch threshold) were subsequently used to decide gene family membership as described in Table 2.

**Table 2:** Decision table used to identify gene family members

Hit is:	Above Threshold	Below Threshold
In Known Cluster	Complete clusters (Keep)	(Reject)
Outside Known Cluster	Singleton cluster (Keep)	(Reject)
	Partial cluster (Keep if cluster representation is ≥50%)	
	Partial cluster (Reject if cluster representation is < 50%)	

The threshold for inclusion was set as the E-value of the last identified Arabidopsis MAP3K in each HMMsearch output. A *known cluster* is any cluster that contains a previously identified MAP3K. A *singleton cluster* was defined as a cluster with only a single gene present (including clusters with multiple transcript variants of the same gene), a *complete cluster* is a cluster where all genes are above the threshold of inclusion, and a *partial cluster* was defined as a cluster with members located both above and below the threshold for inclusion. All Arabidopsis MAP3Ks from the kinome examination were kept. The results of classification decisions (gene identifiers and protein sequences) are available in Additional file 1 (All_MAP3Ks.xlsx).

In a comprehensive examination of the Arabidopsis kinome, Zulawski et al. [1] reported 48 RAFs in Arabidopsis - excluding AT2G43850 as it didn’t pass their threshold for inclusion as a kinase protein – exhibiting only 69.72% similarity to a kinase HMM, below their threshold of 70%. AT2G43850, however, was included as a 49th RAF in the present study as it has recently been shown to be a functionally active RAF-like kinase involved in environmental stress responses [14].

Using this classification methodology, we categorized 108 ZIK, 255 MEKK, and 468 RAF genes. Table 3 gives a comparative layout of all previously and newly identified MAP3Ks; a list of all presently identified MAP3Ks with their longest transcript variants - organized by subfamily - can be found in Additional file 1. 831 MAP3Ks – containing 365 newly identified MAP3Ks - were identified within the seven examined species, including newly identified MAP3Ks within the previously unexamined *G. hirsutum* (215 MAP3Ks) and *Z. marina* (51 MAP3Ks).

**Table 3:** MAP3Ks identified in examined species

ZIK						MEKK				RAF		
Species	Ident.	Publ.	New	Total	Ident.	Publ.	New	Total	Ident.	Publ.	New	Total
*A. thaliana*	11	11	0	11	37	37	0	37	49	49	0	49
*G. max*	24	24	4	28	34	34	23	57	84	90	6	90
*G. hirsutum*	N/A	N/A	27	27	N/A	N/A	52	52	N/A	N/A	136	136
*G. raimondii*	12	12	2	14	22	22	5	27	44	44	26	70
*Z. mays*	6	6	3	9	21	22	7	28	43	45	6	49
*S. lycopersicum*	13	16	0	13	30	33	4	34	36	40	13	49
*Z. marina*	N/A	N/A	6	6	N/A	N/A	20	20	N/A	N/A	25	25
Total				108				255				468

“Identified” (Ident.) column represents all genes identified that were previously published. “Published” (Publ.) column represents previously published MAP3Ks, “New” column shows MAP3Ks identified in this study but not in previous studies, and “Total” column represents all MAP3Ks presently identified.

Consistent with previous reports, the relative size of MAP3K subfamilies is similar to the one in Arabidopsis (12% ZIKs, 38% MEKKs and 51% RAFs), except for *Gossypium* where the RAFs have expanded to 63%. While ZIKs appear to have been correctly identified in previous studies, the current study proposes significant additions to both the MEKK and RAF subfamilies. Notably, the soybean MEKK subfamily has a substantial 68% increase in size compared to previous estimates while *G. raimondii* shows a large 59% increase in comparison to its reported RAF subfamily size. For the newly sequenced *Z. marina*, a total of 6 ZIKs, 20 MEKKs, and 25 RAFs were identified, while the allotetraploid *G. hirsutum* was found to encode 27 ZIKs, 52 MEKKs, and 137 RAFs - approximately double the currently identified MAP3Ks in its diploid progenitor *G. raimondii*.

We estimate that the integrative methodology has a high accuracy in assigning kinase families, as 96.1% of previously identified MAP3Ks were also currently identified. The majority of currently excluded yet previously identified MAP3Ks were located in partial clusters rejected by our decision method. These excluded sequences were classified in partially complete OrthMCL clusters with not enough agreement with the HMM search results (less than 50% of the cluster members were detected above the cut-off threshold in the HMM output). For example, some previously identified MAP3Ks in *S. lycopersicum* [8] including Solyc10g079130 (*SlMAPKKK7*6) -labeled CDPK14 in PANTHER [15] - and Solyc01g005030 (*SlMAPKKK1*) -classified as a transmembrane protein in PANTHER- showed both weak similarity to query HMM models and clustered in unselected orthoclusters; Additional file 2 (MAP3K_excluded) details all previously identified MAP3Ks rejected by our decision method. On the other hand, 90% (90/100) of the newly identified genes within examined species were found within clusters containing at least one previously identified MAP3K gene. While a more dynamic threshold of membership inclusion that takes into account motif and evolutionary analyses can be used to better resolve the most divergent members in new genomes, the presented gene family classification depicts a robust estimation of all MAP3Ks in the seven species examined.

### Sequence motif analysis

Following initial gene family identification, conserved sequence motifs associated with subdomain VIII of MAP3K kinase domain were verified; this subdomain has been shown to play a major role in kinase peptide substrate recognition [16]. Although variations exist in motif conservation, all 108 presently identified ZIKs were found to have the GTPEFMAPE(L/V/M)(Y/F/L) motif conserved with a *p*-value < 0.0001 as calculated by FIMO. Further examination revealed that 246/255 MEKKs were found to have the G(T/S)Px(F/Y/W)MAPEV motif and 457/468 identified RAFs were found to have the GTxx(W/Y)MAPE motif similarly conserved. MAP3Ks lacking characteristic conserved sequence motifs include both previously known and newly identified MAP3Ks: 4 known and 5 new MEKKs and 5 known and 6 new RAFs. The MEKK and RAF genes not selected by FIMO analysis at the significance level of 0.0001 were found to include more divergent motifs; reducing the stringency of FIMO searches to include *p*-values < 0.001 resulted in only 1 known and 4 new MEKKs and 1 known RAF remaining without the presence of a detectable, diverging motif (Additional file 3).

We also calculated the FIMO scores of the previously identified MAP3Ks rejected by our decision (Additional file 2, table MAP3K_excluded_FIMO_scores). We detected the conserved motifs in the three excluded ZIKs from S. lycopersicum, in one MEKK from S. lycopersicum and in 8 excluded RAFs, indicating the reason for their previous classification as MAP3Ks despite divergence from other members of their families. Altogether these indicate that another decision factor for gene family membership can be based on the results of the conserved motif search, in addition to homology searches described in our method. However conserved motifs associated with kinase gene families exhibit similarities which reduce their discrimination power for gene family classification.

Conserved domain analysis revealed that while all ZIKs and MEKKs were found to encode only a conserved kinase domain, over half of all examined RAFs displayed secondary domains including Ankyrin repeat regions (12%), PB1 domains (13%), PAS domains (7%), ACT domains (11%) and EDR1 domains (19%). Additional file 4: Figure S1, S2, and S3 contain cladograms for all identified ZIKs, MEKKs, and RAFs respectively, with representations of polypeptide sequences and relevant functional motifs highlighted beside sequence identifiers. Of the 831 examined MAP3Ks only a single RAF kinase returned an unexpected secondary domain; the newly identified Gohir.D06G196600 (which also encodes a PAS domain) was predicted to encode a C-terminal truncated COG3942 superfamily domain. Interestingly, all RAFs with a divergent RAF sequence motif were found to encode secondary domains associated with suspected substrate recognition functions. Eight of the eleven MAP3Ks containing a divergent RAF sequence motif were found to encode Ankyrin Repeat domains widely known to mediate protein-protein interactions [17], while the remaining three RAFs displayed EDR1 domains which have shown to mediate protein-protein interactions in EDR1 [18].

### Phylogenetic analysis

**Fig. 2:**
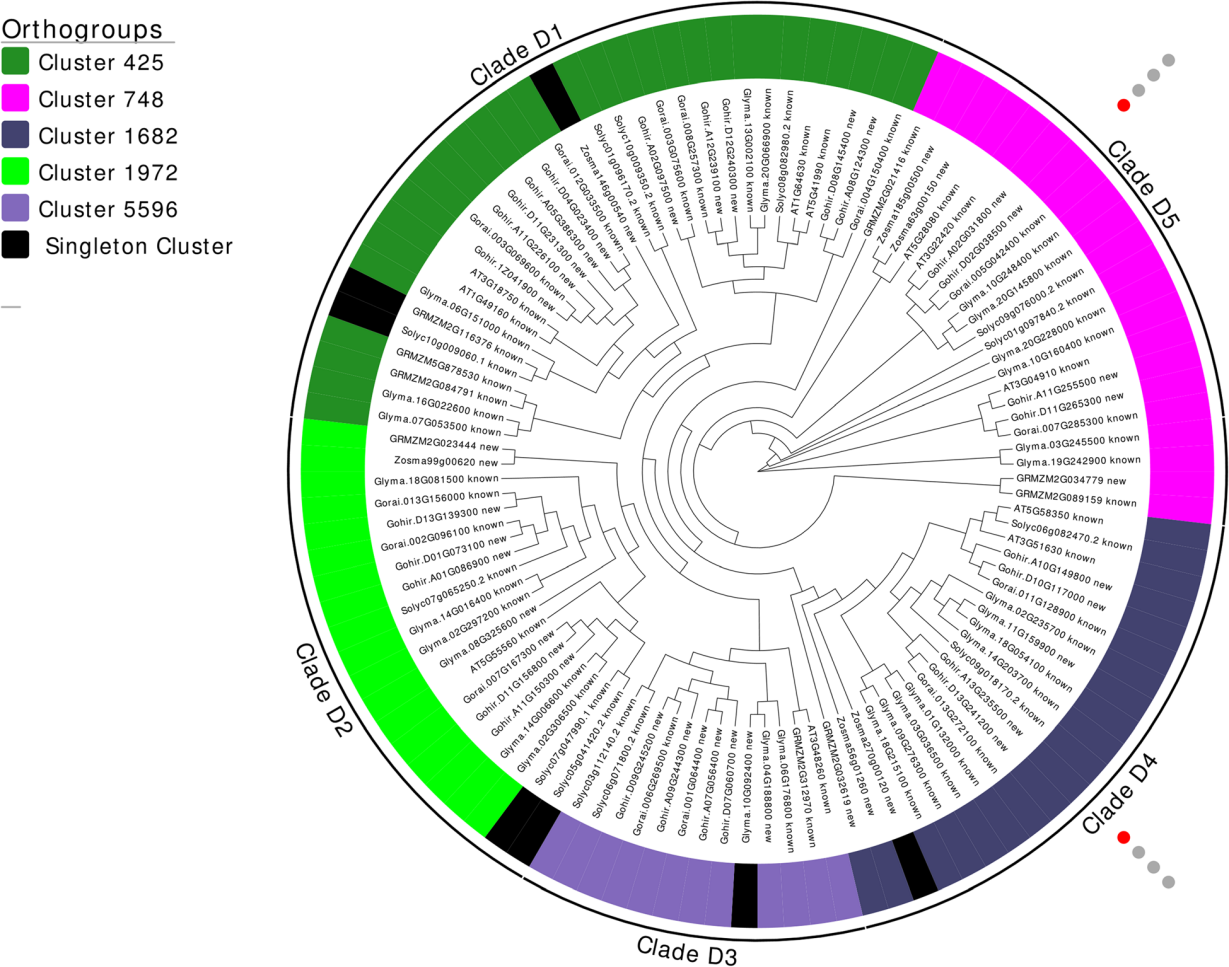
A circular cladogram of ZIK subfamily in seven examined species. OrthoMCL predicted orthogroup clusters are depicted by colored stripes beside sequence identifiers. Significantly differentially regulated genes for *Gossypium hirsutum* are depicted with circles on the outer perimeter where blue circles indicate significant downregulation, red circles significant upregulation, and grey circles are not differentially regulated. Circles represent 1) cold stress, 2) heat stress, 3) drought stress, and 4) salt stress from innermost - > outermost circle

Maximum likelihood trees were annotated with orthogroup identifiers generated by OrthoMCL. Figure 2 depicts a circular cladogram of all presently identified ZIKs. While previous studies [4] have predicted four major clades within the ZIK subfamily - indicative of four ancestral ZIK genes - the current study supports the existence of 5 distinct clades with five major ZIK genes present before the split of monocots and dicots. The five ZIK clades contain at least a single representative gene from at least five of the seven plant species examined – including at least one monocot and one dicot - and clustered well into the five major orthogroups depicted in Fig. 2.

**Fig. 3:**
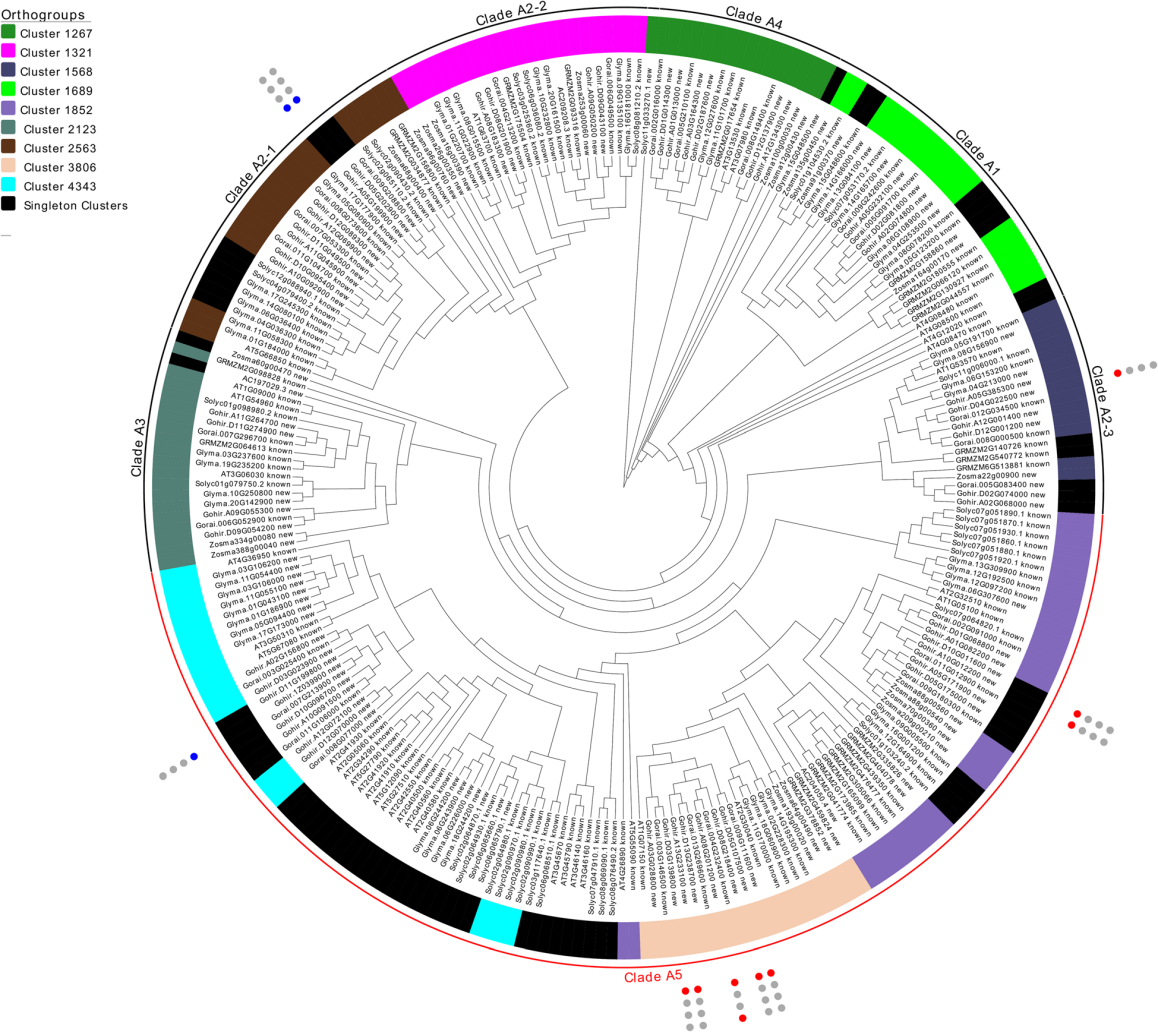
MEKK subfamily of seven plants. Orthogroup labeling is represented by colored stripes beside leaves. Significantly differentially regulated genes for *Gossypium hirsutum* are depicted with circles on the outer perimeter where blue circles indicate significant downregulation, red circles significant upregulation, and grey circles are not differentially regulated. Circles represent 1) cold stress, 2) heat stress, 3) drought stress, and 4) salt stress from innermost - > outermost circle. Clade A1, A3, and A5 are expansions of their previously defined clades defined by MAPK group in 2002. Clade A2 has been split into three subclades (A2–1, A2–2, and A2–3) based on orthogroup cladding. The largely expanded clade A5 (in red) is newly proposed - residing between clades A2 and A3

MEKKs were also previously classified into four major subclades. Orthogroup clustering and a more comprehensive phylogenetic examination support the addition of a 5th MEKK clade (Clade A5 in Fig. 3). The new clade contains representatives from all species examined and contains about half of all presently identified MEKKs (118/255 MEKKs; 67 known and 51 new). Orthogroup cladding supports the existence of 9 ancestral MEKKs before the split of monocots and dicots, although clade A5 appears to have expanded significantly resulting in the formation of numerous paralogs within all examined species.

**Fig. 4:**
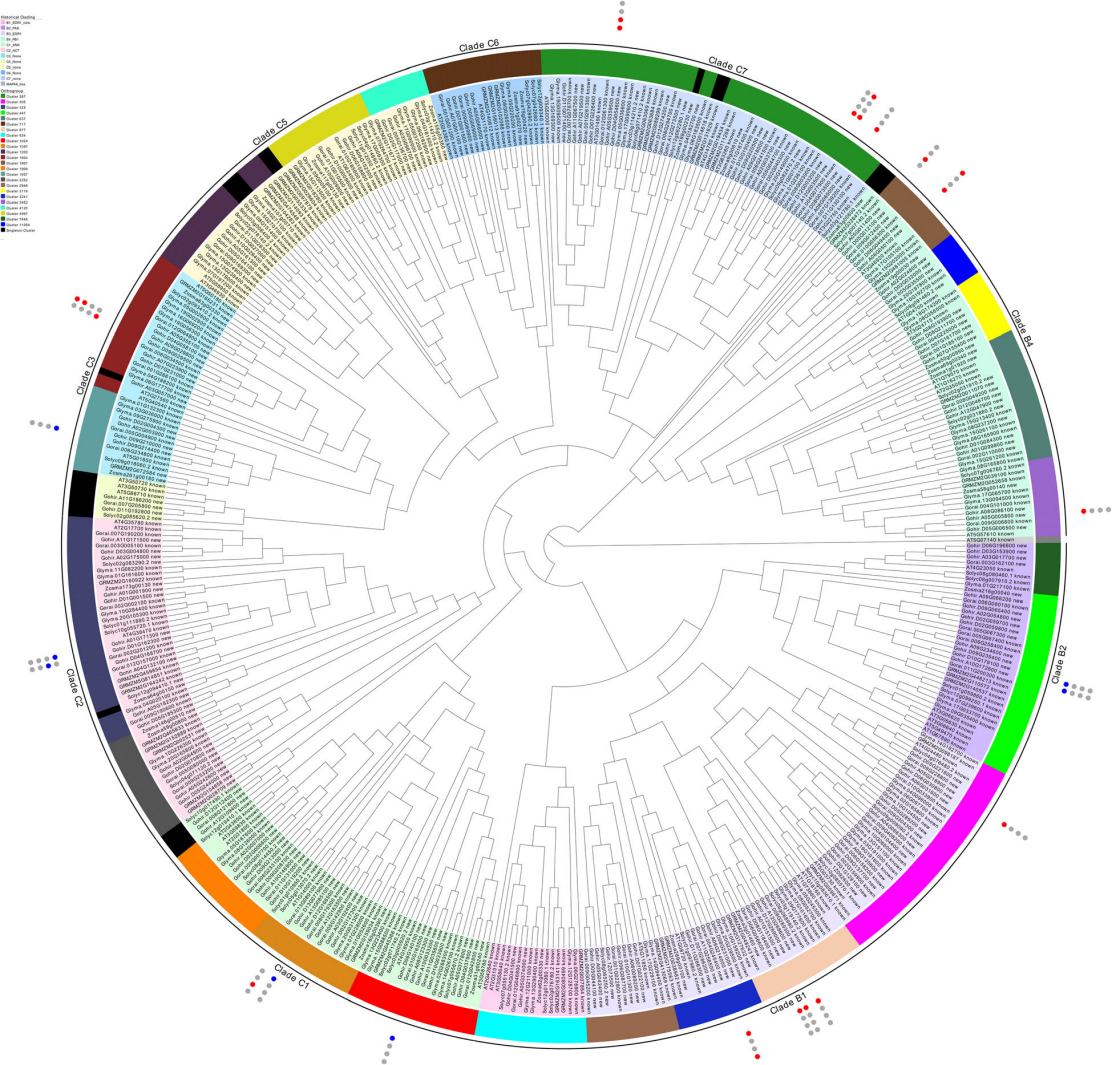
RAF subfamily within seven plants. Significantly differentially regulated genes for *Gossypium hirsutum* are depicted with circles on the outer perimeter where blue circles indicate significant downregulation, red circles significant upregulation, and grey circles are not differentially regulated. Circles represent 1) cold stress, 2) heat stress, 3) drought stress, and 4) salt stress from innermost - > outermost circle. Clade B3 is a combination of previously defined clades B1 and B3. Clade C3 is a combination of previously defined clades C3 and C4. Clades B2, B4, C1, C2, C5, C6, and C7 are analogous to previously established clades. Leaves are colored according to the previously proposed cladding

A comparison between previously suggested RAF cladding and the present orthogroup cladding reveals good conservation of major clades. However, three major refinements should be noted from the current analysis. First, while all RAF clades appear to have undergone various degrees of expansion within the examined plant species, the previously proposed clade C4 – containing ATN1-like kinases – appears to lack this expansion and is found to be constituted of only seven members, three of which are in Arabidopsis with no representation in monocots. This lack of expansion suggests a merging of clade C4 with its phylogenetic neighbor clade C3, which displays an appropriate pattern of expansion across the examined species. The two merged clades are represented by the new clade C3 in Fig. 4. Second, recent domain classification in the CDD/SPARCLE database [19] indicates EDR1 functional domains in both clades B1 and B3, whereas previously EDR1 domains were only detected in clade B3. As the two clades are phylogenetic neighbors, their merger results in the combination of functionally and evolutionarily similar RAFs into a single EDR1 clade. In the present analysis, the previously proposed clades B1 and B3 are combined into the single clade B3. Lastly, AT5G07140 – classified as a MAP3K-RAF following an extensive kinome examination in *Arabidopsis thaliana* [1] – is a distinct outlier among the currently identified RAF genes. Orthogroup classification places AT5G07140 in a cluster containing AT5G58520, a predicted MAP4Ks, and no other members of this cluster were classified as RAFs in the HMM search output. Since threre is no support in our analysis to include it with the RAF family, it is excluded from the currently proposed clades and instead is used to root the RAF subfamily phylogenetic tree.

### Gene duplication and collinearity analysis

**Fig. 5:**
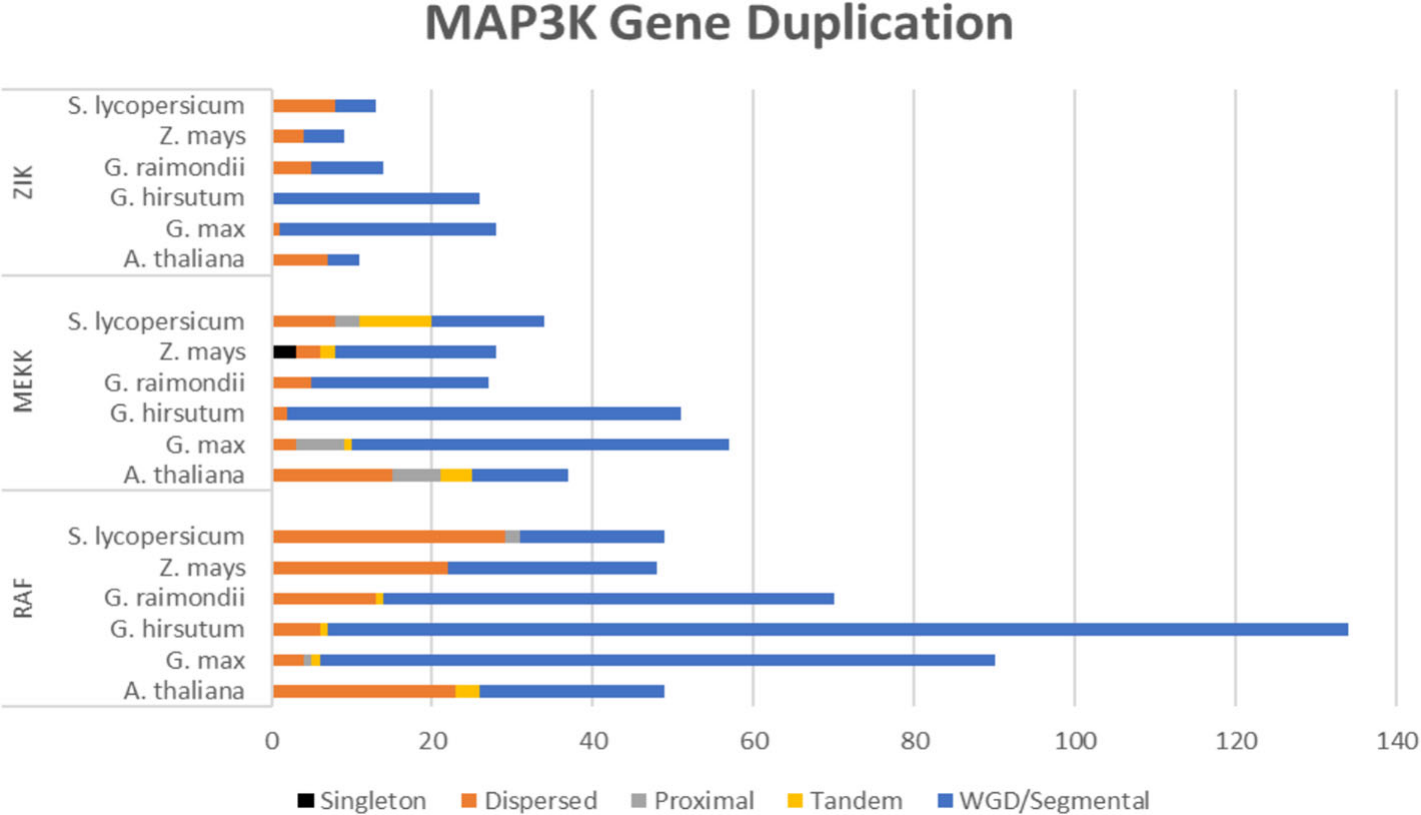
Gene duplication analysis of MAP3Ks. Examination of how gene duplication events have contributed to a MAP3K family expansion in 6 plants (*Zostera marina* was removed as its genome lacks sufficient assembly)

Gene duplication events within six of the seven species examined (*Zostera marina* was removed as its genome lacked a sufficient level of assembly) were explored by locating physical locations of MAP3K genes on individual chromosomes. Figure 5 depicts how gene duplication events have expanded MAP3K subfamilies within the examined plants. WGD/segmental (74.1%) and dispersed (20.4%) gene duplication events were primarily responsible for subfamily expansions. Additional file 5 details predicted duplication origins for each examined MAP3K. As expected, *G. hirsutum*, *G. max,* and *G. raimondii* consistently encoded the largest number of MAP3Ks among the species examined as they also represent the species that have undergone the most recent major gene duplication events [20].

**Fig. 6:**
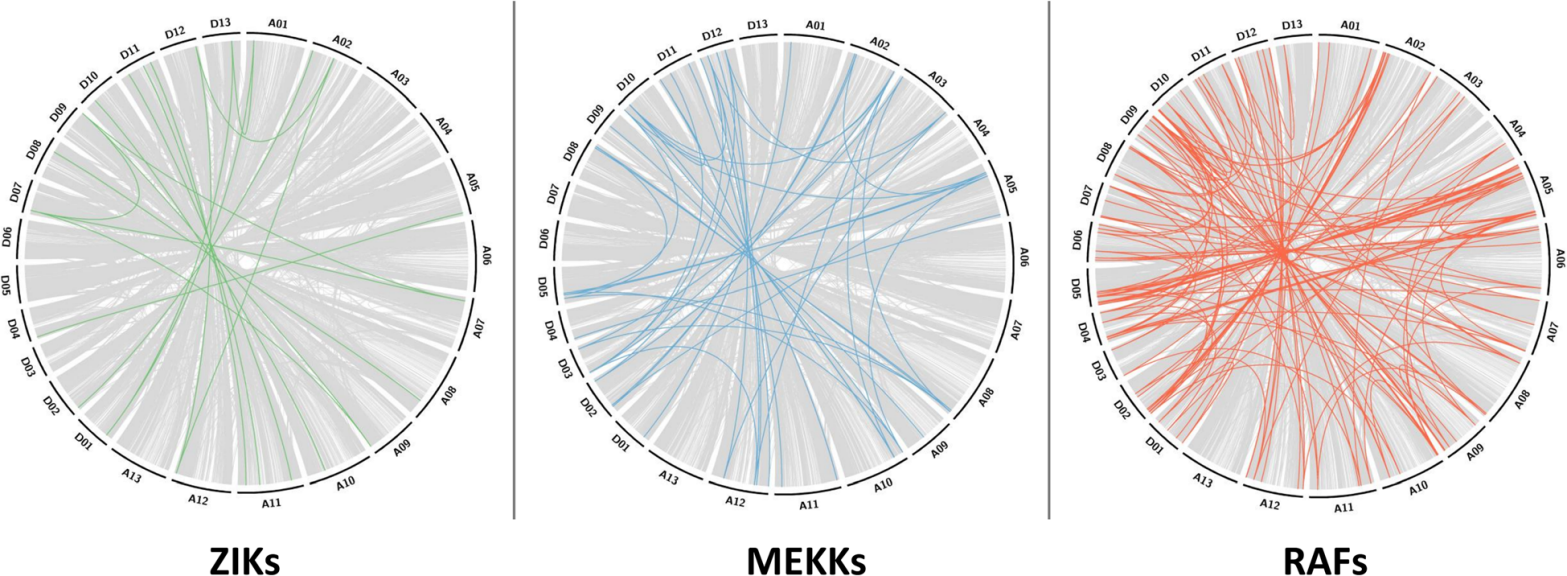
Circular collinearity plots of *Gossypium hirsutum* MAP3Ks. Subfamily-specific collinear relationships are highlighted atop a grey genomic collinearity background. While ZIKs and MEKKs display consistent collinearity between the two subgenomes, RAFs have preferentially expanded within the D subgenome of RAFs

Collinearity is a specific form of synteny requiring conserved gene order. Of the 215 MAP3Ks identified in *Gossypium hirsutum,* 211 were mapped to major chromosomes allowing for the detection of 194 collinear relationships. Collinear relationship of 133 RAFs, 43 MEKKs, and 18 ZIKs are displayed in Fig. 6. As expected, the collinearity observed within the MAP3K family in *G. hirsutum* can primarily be attributed to its allopolyploid genome as 70.6% of collinear blocks were found between the A and D subgenome – representative of gene duplicates arising from its recent allopolyploidy. Although similar distributions of collinearity were observed within ZIKs and MEKKs, RAFs appear to have preferentially expanded within the D subgenome with almost twice (1.93x) as many collinear relationships present exclusively within the D subgenome compared to the A subgenome (Additional file 6). These results support previous observations that the D subgenome has undergone multiple rounds of duplication and chromosomal rearrangements [21] and indicate that the large expansion of RAFs in *G. hirsutum* compared to other plants, likely resulting from its recent allopolyploidy.

*Ka/Ks* ratios were calculated to examine how MAP3Ks have diverged following duplication; *Ka/Ks* < < 1 generally indicates negative or purifying selection, *Ka/Ks* = 1 indicate neutral selection, and *Ka/Ks* > > 1 indicates positive selection. The *Ka/Ks* of all *G. hirsutum* MEKKs and all but one ZIK and one RAF were substantially lower than 1, with average *Ka/Ks* values of 0.31, 0.36, and 0.28 reported for ZIKs, MEKKs, and RAFs, respectively. These results indicate a strong selection bias towards gene function conservation in MAP3Ks following gene duplication events and are thought-provoking as they suggest conservation of signaling pathways in which MAP3Ks operate across the examined genomes [22, 23]. The observed values are consistent with previous findings that species tend to preferentially retain gene duplicates involved in signal transduction and stress response following duplication events, increasing their environmental robustness and potential for specific adaptations [24].

### Transcriptome analysis

Patterns of gene expression in *Gossypium hirsutum* were explored by querying the cotton functional genomics database CottonFGD [25] with the newly identified MAP3K genes. 2 ZIK, 11 MEKK, and 23 RAF MAP3Ks were found to exhibit at least a 50% change in expression in response to at least one biotic/abiotic stress in *Gossypium hirsutum*. These differentially expressed MAP3Ks are labeled in Figs. 2, 3, 4; further examination revealed that 69.4% of identified *Gossypium hirsutum* MAP3Ks displayed at least a moderate 20% change in regulation in response to at least one of the four examined factors (Additional file 7: Table S1). Cold stress was found to induce the largest differential expression among MAP3Ks with 91.7% of all significantly differentially expressed genes showing differential expression in response to cold stress treatment. Interestingly, MEKKs were found to consistently display the largest increases in differential expression with 4 of the top 5 upregulated MAP3Ks classified as MEKKs - while RAFs consistently displayed the largest downregulation – 5 most downregulated MAP3Ks.

Of the 11 identified differentially regulated MEKKs, the newly identified clade A5 was found to contain seven upregulated, and one downregulated MEKK – strongly indicating that gene expansions within clade A5 resulted in several paralogs which might be critical participants in cold stress response. An examination of the many-to-many orthologous relationships within this clade revealed support for this claim. The closest Arabidopsis orthologs for the five upregulated *Gossypium hirsutum* genes in cluster 3806 in Fig. 3 were found to be AT1G07150 and the *NPK1*-like AT2G30040; previous transcriptomics analyses have revealed that both were rapidly and significantly up-regulated during cold stress responses [26]. AT2G32510, the ortholog of Gohir.D10G011600 and its homeolog Gohir.A10G012200, was similarly found to be upregulated in response to cold-stress [27]. Interestingly, the only MEKK downregulated in clade A5 was Gohir.1Z039900. Gohir.1Z039900 displays a many-to-many orthologous relationship with AT3G50310 (*AtMAPKKK2*0), AT4G36950 (*AtMAPKKK2*1), and AT5G67080 (*AtMAPKKK19*). While *AtMAPKKK19* was upregulated during drought and heat stress [28], functional characterization of *AtMAPKKK20* revealed cold stress-induced upregulation [29] indicative of neofunctionalization resulting from diverging homology within some of the paralogs in Clade A5.

C-clade RAFs appear to be involved in osmotic stress sensitivity. *G. hirsutum* RAFs within Clades C1, C2, C3, and C7 displayed significantly differential responses to osmotic stress conditions compared to non-stressed controls (Fig. 3). Although not significantly upregulated, Gohir.A10G009900 located in Clade C5 was previously identified as a *MAPKKK* involved in drought, salt, and cold stress response [30] and found to be moderately upregulated (20.8%) in response to cold stress and downregulated in response to drought stress (22.5%) in the present transcriptome analysis. Clade C6, likewise, contains three *G. hirsutum* genes displaying moderate differential regulation in response to the examined osmotic stresses. Previous functional characterizations of At1g62400 (*HT1*; Clade C5), At4g18950 (*BHP*; Clade C1), At2g17700, At4g35708, At4g38470 (*STY8*, *STY17*, and *STY46* respectively; Clade C2) and At2g43850 (*ILK1*; Clade C1) further support the role in osmotic stress control for C-clade RAFs [14, 31–33]. Gohir.A08G065000 in Clade C7 was functionally characterized as *GhMAP3K6*5 and found to be involved in pathogen and heat stress susceptibility [34]. *GhMAP3K65* was also identified as highly upregulated during cold stress treatment. Gohir.D12G274200 in Clade C7 was recently functionally characterized in transgenic *Nicotiana benthamiana*; transgenic plants overexpressing *Gohir.D12G274200* showed increased pathogen susceptibility and improved tolerance to drought and salt stress at the seedling stage [35].

## Conclusion

Our gene family definition method integrating orthologs clustering and profile HMM homology search was in very good agreement with previous large-scale studies on defining gene families in plants. However, significant differences were detected when compared with studies focusing on MAP3K genes in recently sequenced organisms (*S. lycopersicum*, *G. raimondii*, and *G. max*). That may be due to inherent difficulties in using a single homology search method and in defining adequate threshold levels for gene family definition. Here is where the integration of the whole genome with profile-based homology search methods provided an adequate set of rules for gene family definition. Also, conserved motif analysis, phylogenetic analysis, and gene duplication/collinearity analyses allowed for a better definition of gene clades, using evidence from gene family evolution and functional motif conservation. Large changes from the previously reported MAP3K families were found in the expanded subfamily of RAFs (*G. raimondii* and *S. lycopersicum*) but also in the more conserved MEKK subfamily (*G. max*).

Compared to previous estimates, within the re-examined species, newly identified MAP3Ks account for a significant change in MAP3K family size. While previously having gone unreported, the newly identified MAP3Ks consistently display phylogenetic similarity and high sequence homology to known MAP3Ks and encode subfamily specific motifs and functional domains, indicating potential shared functional equivalency. Although current findings provide an accurate assessment of MAP3Ks in seven plant species, improvements in gene family member identification could be achieved with the application of a more dynamically inferred threshold; potentially one defined using a machine learning algorithm.

Significant expansions within the MAP3K gene family have been uncovered following an extensive examination of plant monocots and dicots. These findings allowed for refinement of the previously proposed MAP3K family cladding. A more comprehensive sampling of plant species, extensive ortholog clustering, functional domain characterization, and subfamily specific, HMM-based homology assessments allowed for a robust definition of MAP3Ks.

In the diverging MEKK and RAF subclades, conserved subclade functionality is supported by transcriptomic evidence, recent gene characterization studies, and orthogroup clustering. Clade C RAFs were consistently found to be differentially regulated in response to osmotic stresses – indicating their likely roles in osmotic stress responses; these roles were found to be supported by gene characterization studies of individual MAP3Ks in both *G. hirsutum* and *A. thaliana*. The newly identified Clade A5 also displayed a conserved role in cold stress response, with support from studies in Arabidopsis. A more extensive examination of MAP3Ks is needed to associate functionality with subfamily cladding. Identification of differentially regulated MAP3Ks can be used to detect targets of significant importance in plant stress response.

Gene duplication and collinearity analyses showed that MAP3Ks had expanded primarily due to WGD events. In *G. hirsutum*, collinearity analysis revealed that while good collinearity was maintained between the A and D subgenomes in MEKKs and ZIKs, gene duplications within its D subgenome had an increased contribution to the expansion of RAF subfamily.

The work presented in this study provides an extensive examination of how MAP3Ks have expanded in plants and for the first time establishes the MAP3K gene family in the commercially important *G. hirsutum* as well as the recently sequenced monocot *Z. marina*.

## Methods

### *Sequence retrieval, database construction, and* MAP3K *identification*

To perform multispecies MAP3K analyses, the complete proteomes of *Arabidopsis thaliana* [36], *Gossypium raimondii* [37], *Gossypium hirsutum* [38], *Solanum lycopersicum* [39], *Glycine max* [40], *Zostera marina* [41], and *Zea mays* [42] were retrieved from Phytozome v12 [43]. The sequences were uploaded onto the Cyverse Discovery environment and clustered into orthogroups using the OrthoMCL workflow detailed at (https://pods.iplantcollaborative.org/wiki/pages/viewpage.action?pageId=12881253). Using the default E-value cutoff, the top 300 hits and alignments of each query were retained as input into the OrthoMCL pipeline [44]. The “OrthoMCL v1.4” application was used to cluster orthologs; index mode was set to all, a *p*-value cutoff of 1.5, percent identity cutoff of 0, percent match cutoff of 0, a maximum weight of 350, and an inflation parameter of 1.5 were used for clustering. Orthogroups were generated by querying the output file using “queryOrthoMCL”. Clustered orthogroups are available in Additional file 8.

Subfamily-specific HMMs and HMMsearches were built and run using HMMER 3.1b2 available at http://hmmer.org/. The threshold used for each MAP3K subfamily is defined as the first instance of a transcript variant of the lowest scoring member of a particular subfamily in Arabidopsis. For the present analysis, AT5G28080.1, AT2G40500.1, and AT5G07140.1 were used for ZIKs, MEKKs, and RAFs with E-values of 3.40E-107, 4.3E-66, and 4.10E-79 respectively. Previously published subfamily members from *Arabidopsis thaliana* [1], *Glycine max* [9], *Gossypium raimondii* [5], *Solanum lycopersicum* [8], and *Zea mays* [3] were used in order to compare how well the decision tree performed in selecting appropriate subfamily members and are represented in “Published” columns of Table 3. Generated HMMs and HMMsearch results for each subfamily are available in Additional file 1. Additional file 9 contains an analysis of partial clusters.

### Sequence motif analysis

Conservation of subfamily specific sequence motifs was performed using FIMO, part of the MEME suite of tools; unique hits in individual proteins with a *p*-value < 0.0001 were associated with motif conservation [45]. Default parameters were used to query NCBI’s Conserved Domain Database (CDD) search tool’s v3.16 database to identify conserved domain motifs [45]. All FIMO predicted hits are available in Additional file 10.

### Phylogenetic analysis

Multiple sequence alignments of subfamily specific MAP3Ks were performed using MUSCLE [46]. Maximum likelihood trees for all alignments were built using MEGA v7.0.26 [47]. Best fit models were predicted using the model prediction tool in MEGA, and maximum likelihood trees were built with default parameters and supported by 200 bootstrap replicates. Tree visualization was generated using Evolview [48]. Cladding for all trees was based on orthogroup clustering generated from OrthoMCL and by using suggested cladding from the MAPK group (MEKKs and RAFs) [11]; all depicted major orthoclusters contained at least 5 of the 7 examined species. All trees are available online at http://120.202.110.254:8280/evolview/#shared/SibKukloHk/723.

### Gene duplication/collinearity analyses

Gene duplication and collinearity analyses were performed using MCScanX using GFF3 files retrieved from Phytozome [49]. Genes that lacked placement on major chromosomes were excluded from examination. Collinearity circle plots were generated using Circos v0.69 [50].

### Transcriptome analysis

FPKM normalized gene expression data for *Gossypium hirsutum* (NAU) was downloaded from CottonFGD [25]. Sequences had to be migrated between JGI and NAU datasets using BLAST. Query sequences from JGI were BLASTed against NAU sequences with unique best hit matches kept for further analysis; if a query had a duplicate hit, the hit on the same subgenome chromosome was used if no conclusive hit was found the gene was removed from the further analysis). Gene expression data for 22 ZIK, 47 MEKK, and 124 RAFs were identified in *Gossypium hirsutum*. For each gene, the log2(FPKM+ 1) of each stress treatment (cold stress, heat stress, drought stress, and salt stress) was subtracted from the log2(FPKM+ 1) of the control treatment to calculate the log2(Fold Change) in each treatment. Genes that displayed 0.5 > Fold change > 1.5 were labeled as significantly differentially expressed in appropriate cladograms.

## Additional files

Additional file 1: Zipped file containing two subfolders and an excel spreadsheet of all identified MAP3Ks with their longest transcript variants. The “HMM_results” file holds the HMMsearch output files for each MAP3K subfamily with relevant orthogroups identification highlighted. The “HMMs” folder holds the HMMs used for each MAP3K subfamily. (ZIP 3956 kb)

Additional file 2: Table describing previously identified, presently excluded MAP3Ks and why they were excluded along with FIMO analysis of excluded hits. (XLSX 19 kb)

Additional file 3: Table containing motif conservation in MEKKs and RAFs with lowered FIMO stringency. (XLSX 46 kb)

Additional file 4: **Figure S1**. Cladograms of identified ZIKs. Image of maximum likelihood tree of ZIKs beside representation of polypeptide sequence with major functional domains identified. **Figure S2**. Cladograms of identified MEKKs. Image of maximum likelihood tree of MEKKs beside representation of polypeptide sequence with major functional domains identified. **Figure S3**. Cladograms of identified RAFs. Image of maximum likelihood tree of RAFs beside representation of polypeptide sequence with major functional domains identified. (ZIP 14020 kb)

Additional file 5: Table containing McScanX predicted gene duplication origins for MAP3Ks in examined species. (XLSX 24 kb)

Additional file 6: **Table S1**. Table containing the *Gossypium hirsutum* transcriptome analysis results. The first sheet has all genes that display at least a 50% change in transcription compared to a control. The second sheet highlights all genes that show at least a 20% change in regulation compared to a control. (XLSX 35 kb)

Additional file 7: Table containing collinear relationships in G. hirsutum as predicted by McScanX. (XLSX 61 kb)

Additional file 8: OrthoMCL generated clusters for all proteins in 7 plant species. (TXT 11283 kb)

Additional file 9: Examination of partial clusters flanking thresholds set for each MAP3K subfamily. (DOCX 13 kb)

Additional file 10: Table containing FIMO predicted motif conservation hits in all examined MAP3Ks. (XLSX 55 kb)

## Abbreviations

BLAST: Basic Local Alignment Search Tool
HMM: Hidden Markov Model
MAP3K: Mitogen-activated protein kinase kinase kinase
MEKK: MEKK kinase subfamily
RAF: RAF kinase subfamily
ZIK: ZIK kinase subfamily
