# Supplementary figures and images for: Multispecies genome-wide analysis defines the MAP3K gene family in Gossypium hirsutum and reveals conserved family expansions
Source: BMC Bioinformatics. 2019 Mar 14;20(Suppl 2):99. doi: 10.1186/s12859-019-2624-9 (PMC6419318; doi:10.1186/s12859-019-2624-9)

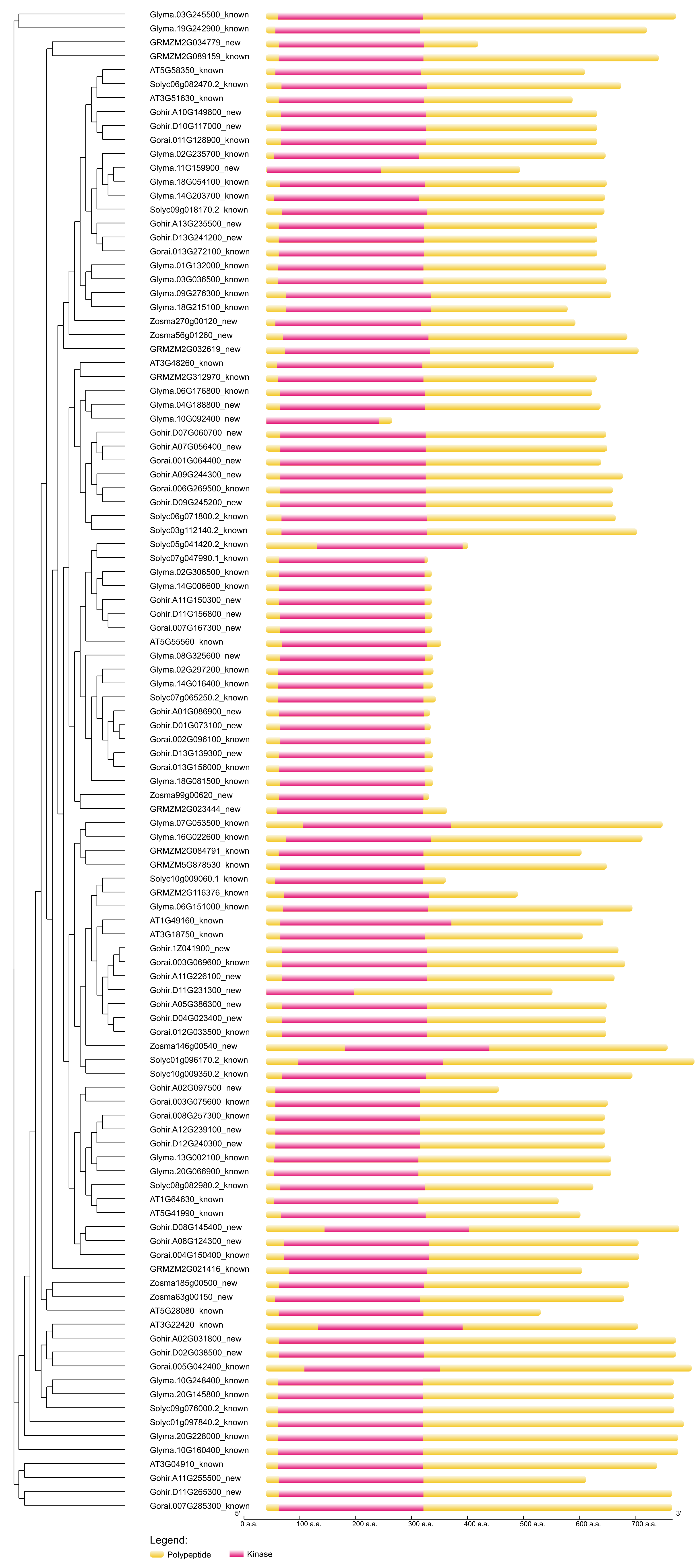

Supplement: Supplementary file 4 — Figure S1. Cladograms of identified ZIKs. Image of maximum likelihood tree of ZIKs beside representation of polypeptide sequence with major functional domains identified. Figure S2. Cladograms of identified MEKKs. Image of maximum likelihood tree of MEKKs beside representation of polypeptide sequence with major functional domains identified. Figure S3. Cladograms of identified RAFs. Image of maximum likelihood tree of RAFs beside representation of polypeptide sequence with major functional domains identified. (ZIP 14020 kb) [file 12859_2019_2624_MOESM4_ESM.zip › Figure S1.tiff]

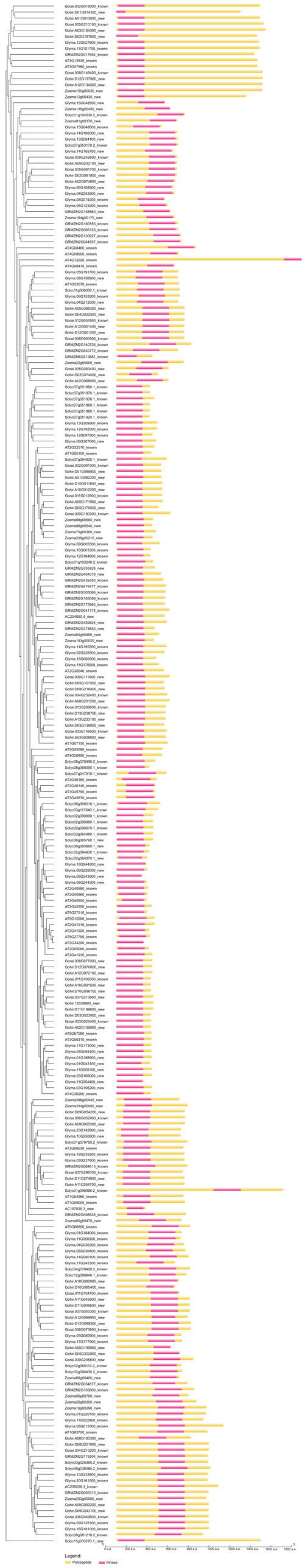

Supplement: Supplementary file 4 — Figure S1. Cladograms of identified ZIKs. Image of maximum likelihood tree of ZIKs beside representation of polypeptide sequence with major functional domains identified. Figure S2. Cladograms of identified MEKKs. Image of maximum likelihood tree of MEKKs beside representation of polypeptide sequence with major functional domains identified. Figure S3. Cladograms of identified RAFs. Image of maximum likelihood tree of RAFs beside representation of polypeptide sequence with major functional domains identified. (ZIP 14020 kb) [file 12859_2019_2624_MOESM4_ESM.zip › Figure S2.tiff]
