# Supplementary material for: Multispecies genome-wide analysis defines the MAP3K gene family in Gossypium hirsutum and reveals conserved family expansions
Source: BMC Bioinformatics. 2019 Mar 14;20(Suppl 2):99. doi: 10.1186/s12859-019-2624-9 (PMC6419318; doi:10.1186/s12859-019-2624-9)
Supplement: Supplementary file 9 — Examination of partial clusters flanking thresholds set for each MAP3K subfamily. (DOCX 13 kb) [file 12859_2019_2624_MOESM9_ESM.docx]

*Format: <partial cluster> - % of cluster represented in HMMsearch output represented above threshold followed by relevant information about particular members of cluster/why cluster was examined.*

**ZIKS**

425- 96.9% (62/64 cluster members) 2 hits below threshold have no transcript variants above threshold. Both have good E-values and should potentially be included as ZIKs.

637 – cluster exclusively below threshold, only included for examination as Solyc02g031860.2.1 was previously identified as a ZIK. It is better placed (and included in present study) as a RAF.

748- 96% (48/50) represented cluster members). all genes represented above threshold

5596- 89% (16/18); all genes represented above threshold

71716- singleton; previously id’d Solyc02g087590.1 is potentially zik w/ panther and motif conservation support. Excluded from presently examined genes

**MEKKs**

420- single hit for mekks, previously identified Solyc01g005030.2.1 is actually a transmembrane domain containing protein kinase – not a mekk/map3k.

1267 – 89.7% (35/39); of the 4 genes below, 2 have transcript variants above threshold, while 2 genes GRMZM2G305321 and GRMZM2G084586 are uniquely below the threshold. They also have relatively lower E-values compared to hits above threshold with E-values of 7.00E-47 and 6.30E-15 respectively.

2123 – 93.3% (28/30); all genes represented above threshold

8416 – 30.8% (4/13); all hits below threshold excluded. Only Solyc02g064930.1.1 previously identified as mekk

15247 – 66.7% (4/6); 2 members below cluster were not included in 2014 kinome examination; included all hits above threshold, excluded ones below as ones above were included in kinome classification.

20106 – 75% (3/4); 4 tomato genes within cluster; single gene below threshold excluded; potential for inclusion as map3k

72947 – exclusively below threshold, Solyc04g064590.1.1 previously identified as mekk

77245 – exclusively below threshold, Solyc12g005360.1.1 previously identified as mekk

**RAFs**

287 – 87.3% (69/79); all genes represented above threshold

305 – 89.7% (70/78); of those below threshold, 5 had transcript variants above threshold; Gohir.A05G288000.1, Gohir.A01G139900.1, and Glyma.02G215300.1 (known) were excluded, but may be rafs.

325- 89.5% (68/76); all but Gohir.D11G178400.1 have transcript variants above threshold.

447 -98.4%(62/63); all genes represented above threshold

600 – contains previous identified Solyc10g085670.1.1; cluster never represented above threshold

637 - 92.5(49/53); one gene represented above threshold; one gene with 3 transcript variants found exclusively below threshold (Glyma.08G237100) new

717 – 82.4% (42/51); all but one gene – GRMZM5G852329 (previously unexamined)– have transcript variants above threshold, promising e value

877 – 93.5% (43/46); all genes represented above threshold

924 – 86.7% (39/45); all but Solyc12g013970.1.1 (new) represented above threshold

1024 – 72.1% (31/43); all genes represented above threshold

1097 – 85.4% (35/41); all but Zosma10g00140.1 represented above threshold

1200 – all genes represented above threshold

1267 – exclusively below threshold;

1804 – 97% (32/33); all genes represented above threshold

1807 – 78.8% (26/33); all but GRMZM2G145360 (previously unexamined) found above threshold

1908 – 56.3% (18/32); all but GRMZM2G007466 and GRMZM5G882078, and Zosma76g00760.1 found above threshold

1957 – 96.9% (31/32); all genes represented above threshold

2252 – 83.3% (25/30); all genes represented above threshold

3116 – 88% (22/25); all genes represented above threshold

3241 – 83.3% (20/24); all but GRMZM2G044180 (new) found above threshold

7448 – 93.3% (14/15); all genes represented above threshold

7496 –6.7% (1/15); single Arabidopsis gene above threshold; it is the map4k like hit

12478 – 77.8% (7/9); all genes represented above threshold

12796 – exclusively found below threshold

19762 75% (3/4); single Glyma.12G233200.1.p not found above threshold

26303 – 66.7% (2/3); all genes represented above threshold

33941 – exclusively found below threshold

56112 – singleton, found exclusively below threshold
